# The Honeybee Gut Mycobiota Cluster by Season versus the Microbiota which Cluster by Gut Segment

**DOI:** 10.3390/vetsci8010004

**Published:** 2020-12-31

**Authors:** Jane Ludvigsen, Åsmund Andersen, Linda Hjeljord, Knut Rudi

**Affiliations:** Faculty of Chemistry, Biotechnology and Food Science, Norwegian University of Life Sciences, 1430 Ås, Norway; jane.ludvigsen@nmbu.no (J.L.); aan@mycoteam.no (Å.A.); linda.hjeljord@nmbu.no (L.H.)

**Keywords:** honeybee, gut microbiota, gut mycobiota, season

## Abstract

Honeybees represent one of the most important insect species we have, particularly due to their pollinating services. Several emerging fungal and bacterial diseases, however, are currently threatening honeybees without known mechanisms of pathogenicity. Therefore, the aim of the current work was to investigate the seasonal (winter, spring, summer, and autumn) fungal and bacterial distribution through different gut segments (crop, midgut, ileum, and rectum). This was done from two hives in Norway. Our main finding was that bacteria clustered by gut segments, while fungi were clustered by season. This knowledge can therefore be important in studying the epidemiology and potential mechanisms of emerging diseases in honeybees, and also serve as a baseline for understanding honeybee health.

## 1. Introduction

The importance of the gut microbiota (GM) in regulating honeybee (*Apis mellifera*) health has become increasingly evident in recent years. Regarding nutritional and immunological interactions with the host, the bacterial proportion of the GM is most frequently studied [1,2,3,4,5,6], but studies investigating the fungal part of the microbiota and how some fungi interact with honeybee pathogens have recently emerged [7]. In humans, the fungi part of the GM has recently been linked to human infant development [8].

The GM of honeybees harbor a specific set of 8–9 bacterial groups, which all seem to have evolved and adapted to a life in the guts of honeybees, and they are only found in honeybees or in closely related bees such as bumble bees [9]. These bacterial groups aid in nutritional breakdown of pollen and nectar, interact with the immune system, and contribute to pathogen defense in the gut [10]. Likewise, specific fungi show interactions with pathogens in honeybees [11,12], but little is currently known about the role of fungi in the GM of honeybees.

The honeybee gastrointestinal tract (GI) can be divided into four main parts: crop, midgut, ileum, and rectum. These four parts have been shown to harbor unique bacterial species for which metabolic properties have been elucidated [13]. Although detailed information about each bacterial species community changes, little information is available about how the bacterial community changes in regard to outer stimuli. Only a few studies have described how the GM composition changes according to season [14,15] and developmental stages of the host [16,17]. The fact that specific bacteria change during the season is an indication that diet contributes to variation in composition. Yun et al. 2018 [13] found that foragers harbor a different set of fungi than that of nurse bees, indicating that diet is a source of variation in the fungal composition as well. They also found that queen bees carry an overload of one type of fungi (*Zygosaccharomyces*), which is different from nurse bees, indicating that the fungal part of the microbiota, as seen for the bacterial part, has adapted to different lifestyles, which is reflected in the overall fungal microbiota [13].

All previous studies have investigated the microbiota of the total GI (crop to rectum or midgut to rectum), resulting in a lack of information about the microbiota of different gut parts under these scenarios. Since some honeybee pathogens are gut part specific, information about the variation in the microbiota composition in different gut parts is crucial for understanding the microbiota–pathogen–host dynamics in more detail.

Here, we investigate both the bacterial and fungal parts of the GM (by gut parts)—crop, midgut, ileum, and rectum—throughout an entire season (longitudinal), from March until November in adult honeybee workers. Our results can aid in understanding specific gut part-specific interactions and help in the design of later in-depth functional and metabolic studies.

## 2. Materials and Methods

### 2.1. Sampling

The bees were sampled from two neighboring hives located at the Norwegian University of Life Sciences, As, Norway, across seven time points representing before, during and after foraging season. Ten bees where picked randomly from the frame closest to the opening of both hives to represent foragers in April, June, July, and August. In March and November, the bees are not foraging but are clustered together to keep warm, and thus ten bees from both hives were picked from the top of the formed cluster. All bees represent adult bees. All experiments were conducted following Norwegian rules for studies on honeybees [18].

### 2.2. Gut Dissection

Bees were sampled and put on ice to induce chill-coma before dissection. Then, the gut was removed from the bee after we had sterilized the bee on the outside by washing it in 50% ethanol. By pulling out the stinger, the gut from midgut to rectum was removed. The crop was dissected out separately with sterile dissecting tools. Unfortunately, we did not collect crops from bees sampled in March. The gut was cut into its respective parts under a dissection microscope and on sterile microscopy slides (washed with 70% ethanol and 1:10 chlorine) by cutting at the transition areas between the different gut parts (Figure S1). The dissection was performed in a drop of PCR water (VWR, Radnor, PA, USA) and the Malpighian tubes were cleaned off the midgut and left attached to the ileum part. The different gut parts were collected in tubes prefilled with a bead matrix consisting of 0.2 g of each < 106 μM acid-washed glass beads, 0.425–0.600 mm glass beads, and 2 2.5–3.5 mm acid-washed glass beads (Sigma-Aldrich, Darmstadt, Germany) and stool transport and recovery (S.T.A.R) buffer (Roche, Mannheim, Germany). For midguts and rectum samples, 300 μL of STAR buffer were used and 200 µL were used for crop and ileum. The dataset contained four gut parts (crop, midgut, ileum, and rectum) from 20 bees over seven months (except from March, which only contained midgut, ileum, and rectum) for a total of 540 samples. The samples were frozen at −20 degrees before DNA extraction.

### 2.3. DNA Extraction

DNA was extracted from a total of 540 samples, with 484 samples yielding sufficient DNA for further processing. Mechanical lysis was performed using FastPrep (MP Biomedicals, Santa Ana, CA, USA) at 1800 rpm for 40 s, two times, with a 5 min cool-down between runs. The samples were then centrifuged for 5 min at 13,000 rpm and 50 µL supernatant was transferred to a 96-well plate for DNA extraction using the MagTM mini kit (LGC, Middlesex, UK) following manufacturers recommendations. The extraction was performed on the KingFisherTM Flex Magnetic Particle Processor, (Thermo ScientificTM, Waltham, MA, USA). The extracted DNA was frozen at −20 degrees before subsequent PCR/qPCR and Illumina sequencing.

### 2.4. qPCR

Quantification of bacteria and fungi in different gut parts across seasons was performed using qPCR assays targeting the 16S rRNA gene for bacteria and the ITS1 part of the fungal rRNA. Primers targeting the vitellogenin gene of honeybees were used to normalize for possible differences in gut size. Primers used in this study are listed in Table 1. qPCR was performed on LightCycler 480 II (Roche, Mannheim, Germany) using 0.2 μM of forward and reverse primers, 5× HOT FIREPol^®^ EvaGreen qPCRMix Plus (Solis BioDyne, Tartu, Estonia) in 1× concentration, 5 μL gDNA, and adding nuclease-free water (VWR, Radnor, PA, USA) in a total volume of 20 μL. Nuclease-free water (VWR, Radnor, PA, USA) was used as a negative control. PCR conditions for 40 cycles were activated for 15 min at 95 °C, annealing for 30 s at 55 °C and 54 °C for 16S/ITS1 and vitellogenin, respectively, and elongation for 45 s and 30 s for 16S/ITS and vitellogenin, respectively at 72 °C.

Relative copy numbers for 16S rRNA and ITS1 genes were calculated based on standard curves generated from Ampure^®^ XP (Beckham coulter, Brea, CA, USA) purified PCR amplified targets, which were quantified using Qubit^®^ dsDNA HS assay kit (Life technologies, Carlsbad, CA, USA), both methods performed according to the manufacturer’s recommendations. Standard curves were run using 5× HOT FIREPol^®^ Blend Mastermix Ready to Load (Solis BioDyne, Tartu, Estonia) in 1× concentration, with 0.2 µM of forward and reverse primers and 1 µL of gDNA in a total volume of 25 µL. PCR conditions for 30 cycles were as described above for qPCR with an additional 7 min final elongation step at 72 °C.

### 2.5. Illumina Sequencing

Illumina sequencing of the 16S rRNA gene and the ITS1 region was performed using the same primers as described above (table primers), and we used the same library preparation methodology as described in [22]. For the initial PCR conditions, we used the same conditions as for preparation of standard curves, but we only ran 25 cycles for 16S rRNA gene compared to 30 cycles for the ITS1. We indexed the ITS primers with 16 forward and 36 reverse indexes (table ITS index primer sequences). Pooling and preparation of the Illumina library was performed following the 16S metagenomic sequencing library preparation protocol (Illumina, San Diego, CA, USA). Quantification of pooled library was performed by ddPCR, BIO-RAD QX 200™ droplet reader (BioRad, Hercules, CA, USA) and diluted to 7 pM and sequenced on MiSeq using v3 reagents (Illumina, San Diego, CA, USA).

### 2.6. Data Analysis

qPCR copy numbers for all three genes were calculated using SciencePrimer copy-number calculator (Primer 2017), and 16S and ITS were normalized to vitellogenin copy-numbers. Samples in each group that fell outside the 1/3 quartile of the median were removed as outliers.

Illumina fastq files were analyzed as described in [22]. In large, dereplicated, filtered, and generated OTUs in USEARCH v 8, then rarefactioned to 4000 sequences and calculated in QIIME for alpha and beta diversity. Identification of bacterial taxa was done using Green-genes V, and the UNITE (2019) database was used for blast searches for fungal OTUs.

All statistical analyses and plots were conducted in R (v. 3.5.1). Statistical significances were tested using the nonparametric Wilcoxon signed-rank test, with a significance threshold of *p* < 0.05. Dimension reduction of multidimensional data was done using nonmetric multidimensional scaling (NMDS). These analyses were performed using the Phyloseq package (v 1.24.2) in R.

## 3. Results

The relative abundances of bacterial and fungal taxa with respect to gut segment and season are presented in Figures S2 and S3, in addition to Blast-based taxonomic assignments of fungi (Table S1).

### 3.1. Higher Bacterial Abundance than Fungal Abundance in All Gut Parts across the Entire Season

**Figure 1:**
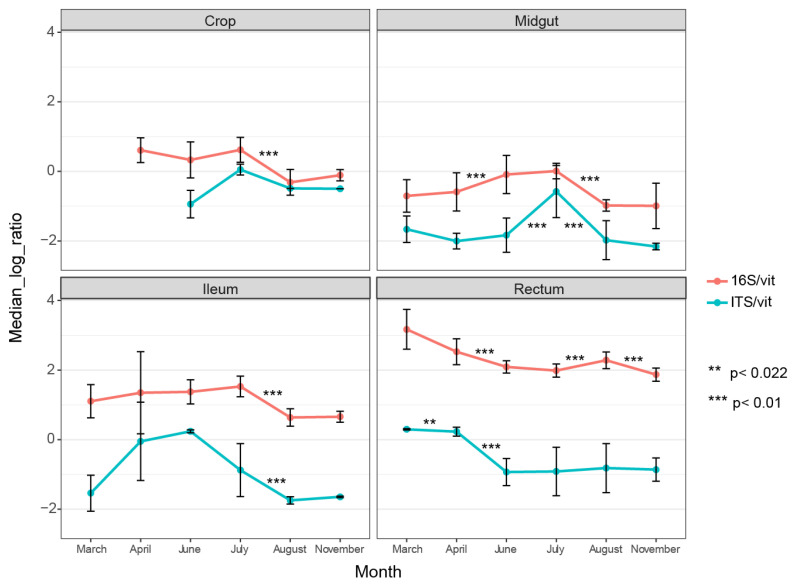
qPCR results showing median relative abundance of bacteria (16S) and fungi (ITS1) across months per gut part. Both 16S and ITS1 gene DNA are normalized against vitellogenin gene DNA. The vitellogenin gene copies were used as a proxy for the weight of the tissue. Error bars represent standard deviations. The asterisks represent statistical significance using the Wilcoxon signed-rank test.

Our dataset enabled us to compare bacterial and fungal abundance in different gut parts and track changes in abundance across the season (before, during, and after foraging). There was a higher bacterial abundance in all gut parts compared to fungal abundance, and this increase became more apparent towards the rear end of the gut (Figure 1). In the rectum, the bacterial load was magnitudes higher on average than the fungal load. Our results are consistent with previous findings of higher bacterial load in the hind gut (ileum and rectum) than in the midgut, as we found that the midgut had the lowest bacterial load across all months.

Interestingly, the fungal abundance in the midgut and ileum was substantially influenced by season, as the copy-numbers fluctuated between neighboring months, creating a peak load in these two gut parts in June and July, respectively (Figure 1). The rectum displayed a different trend, with higher fungal load early in the season. The midgut harbored the lowest fungal load compared to the other gut parts (Figure 1).

### 3.2. Observed Fungal Species Diversity Peaks during Foraging Months

**Figure 2:**
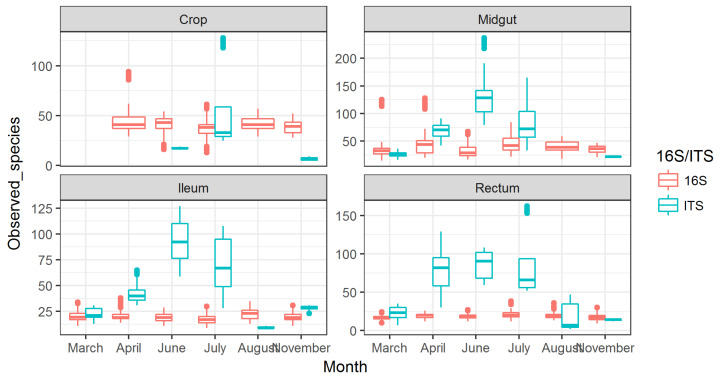
Observed species for bacteria (16S) and fungi (ITS) shown across months for different gut part. The missing data in the figure are due to lack of information for those datapoints. This particularly relates to fungi in crop, as the levels were very low.

Numbers of observed fungal species displayed an increasing trend from April, which peaked in June (4×) and returned to baseline in August (Figure 2). This peak in fungal diversity was observed in midgut, ileum, and rectum, and the highest number was detected in the midgut. The crop showed a less clear peak in July, but the trend was present there as well. The observed fungal diversity due to these peaks was 4× higher than the number of observed bacterial species.

For observed bacterial species diversity, the numbers were 2× higher in the crop than in the other gut parts, although a slight increase in diversity was seen in the late foraging months in midgut samples (Figure 2).

### 3.3. Fungal Communities Cluster by Months and Bacterial Communities Cluster by Gut Parts

**Figure 3:**
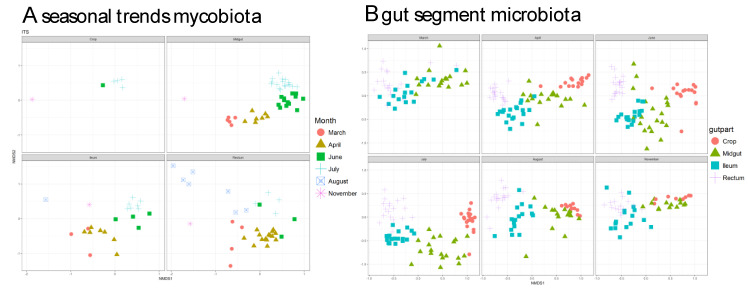
Nonmetric multidimensional scaling (NMDS) plots showing clustering of samples from different gut-parts based on (**A**) mycobiota and microbiota (**B**) composition. The stress values in all cases were < 0.2, suggesting a proper dimension reduction. The reason to present panels connected to gut segment for the mycobiota and season for the microbiota is to highlight the main differences visually.

NMDS plots revealed that the fungal community is highly influenced by season as the different fungal communities each month cluster apart, i.e., fungal communities were not much different between gut parts and the different gut part communities were similar in regard to different months (Figure 3A). This contrasts with the bacterial communities, which clustered by gut parts, i.e., bacterial communities showed highly gut part-specific communities, which were not strongly influenced by season (Figure 3B).

## 4. Discussion

Our main finding was that the gut mycobiota changed with season, while the microbiota was mostly affected by the gut segment. This finding can have major implications for understanding the interaction between bacteria and fungi in honeybee health and disease.

Most diseases have a seasonal trend, but the underlying factors determining the seasonality remain unknown [23]. In temperate climates, honeybees stay inside the hive in winter until the weather is warm enough to fly out. This happens around May, and they continue to forage until late August. Our data suggest that the diversity of fungi species is elevated during foraging months, which was demonstrated for all gut parts. Higher species diversity in foragers has previously been shown by Yun et al. 2018 [13], suggesting that only a few fungi are endemic in honeybees and that a large amount of transient fungi might influence honeybees during summer months. Additionally, we saw that during early months there was more yeast-like fungi in midgut, ileum, and rectum. This could be due to bees being inside the hive for a long time in damp conditions. This is congruent with Yun et al. (2018), who found more yeast in nurse bees, which only stay inside the hive as well. The interplay between yeast, fungi, and bacteria could play a role in managing the yeast load in foraging bees, as we saw that the yeast load decreased as more environmental yeast and bacteria were present. Suggestions of this are found in germ-free mice that are highly susceptible to yeast infections [24], and in honeybees, yeast load seems to correlate with Nosema infection, which often peaks early and late in the year [11].

Mortality of honeybees also shows a clear seasonal trend [25]. Therefore, a factor that has not yet been considered in the mortality models is the major change in the fungal population associated with honeybees. Fungal diseases are commonly opportunistic and difficult to trace [26]. The major seasonal trends discovered here highlight the challenge in understanding fungal associated diseases.

In contrast to fungi, bacteria showed a clear association with gut segments through the season. This indicates that bacteria have a crucial role in maintaining honeybee health [27]. Dissection of the different gut parts, as done in this study, can reveal patterns not possible to detect using whole GI tracts. We found in our study that the midgut was more influenced by season than were ileum and rectum parts, which are usually what most studies investigate [14]. The hindgut comprises >90% of the total bacterial load in honeybees and thus will reflect the variation if the whole GI tract is used, and valuable information about which bacteria might be possible transient bacteria will be lost. There were some bacteria in our study that were mostly in the crop and midgut only in foraging months, which thus could be transient bacteria and not part of the endemic honeybee gut microbiota. This type of detailed (both gut part and different foraging months) information can shed light on previous suggestions that these bacteria are a part of the normal flora because they are commonly found in most bees. In addition to obligate pathogens, the absence of health-promoting bacteria can also lead to disease. Such diseases, however, would be much more difficult to detect, as they cannot be linked to specific bacteria [28]. Thus, diseases connected to lack of function could also have a potential role in explaining honeybee diseases, such as the colony collapse disorder (CCD). Thus, the fight towards the obligate pathogens could lead to simultaneous eradication of bacteria that have essential functions, such as vitamin production [29].

A limitation of our study, however, is that we did not consider the microbiota in the mouth part, which could have a substantial influence on both the micro- and mycobiota in the honeybee gastrointestinal tract [30]. Nor did we do specific measurements of diet [31]. Our study is further limited in that we only investigated one location and two hives. Further studies are therefore needed to generalize our findings. Our study also illustrates experimental issues that need to be considered in honeybee studies. Both season and gut segment had a major impact on the gut myco-/microbiota. Without taking the spatiotemporal information into account, misleading conclusions can be drawn related to the association of the honeybee gut myco-/microbiota with health and disease.

## 5. Conclusions

In conclusion, we have shown major differences connected to gut segment and seasonal associations of the honeybee gut myco-and microbiota. This knowledge can have major implications for honeybee health and disease.

## Figures and Tables

**Table 1:** Primers applied in this work.

Primer	Target	Sequence	Reference
PRK314F	16S rRNA		[19]
PRK806R	16S rRNA		[19]
BITS	ITS1	ACCTGCGGARGGATCA	[20]
B58S3	ITS1	GAGATCCRTTGYTRAAAGTT	[20]
Vitellogenin F	Vitellogenin	GTTGGAGAGCAACATGCAGA	[21]
Vitellogenin R	Vitellogenin	TCGATCCATTCCTTGATGGT	[21]

## Data Availability

Data is contained within the article or supplementary material.

## Supplementary Materials

The following are available online at https://www.mdpi.com/2306-7381/8/1/4/s1, Figure S1: Dissection scheme (a) whole gut, (b) the transition between the middle stomach and ileum, and (c) transition between the ileum and the rectum, Figure S2: Relative abundance of the 20 most abundant fungal OTUs across months per gut part, Figure S3: Relative abundance of the bacterial OTUs (with abundance > 1%) across months per gut part, Table S1: Taxonomic assignments of fungal OTUs by Blast.
