# Supplementary material for: The Honeybee Gut Mycobiota Cluster by Season versus the Microbiota which Cluster by Gut Segment
Source: Vet Sci. 2020 Dec 31;8(1):4. doi: 10.3390/vetsci8010004 (PMC7823634; doi:10.3390/vetsci8010004)
Supplement: Supplementary file 1 [file vetsci-08-00004-s001.pdf]

Supplementary

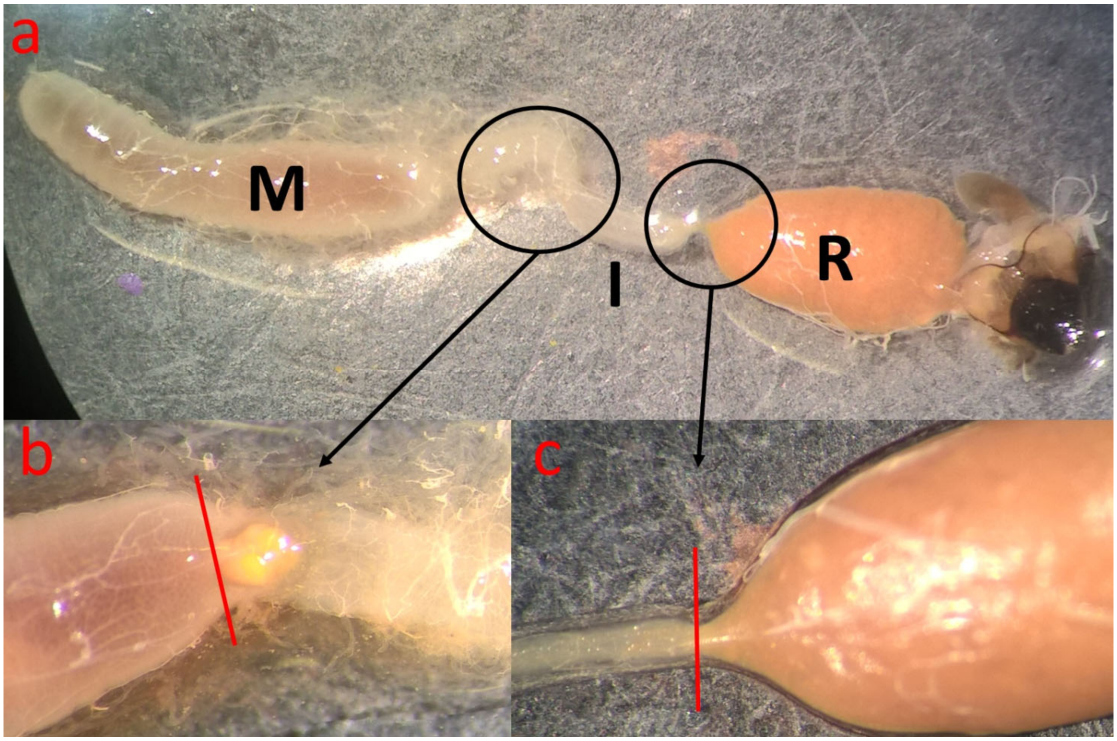

Figure S1. Dissection scheme (**a**) whole gut, (**b**) the transition between the middle stomach and ileum, and (**c**) transition between the ileum and the rectum. Red streaks indicate areas cut. M = Mid stomach, I = ileum and R = rectum.

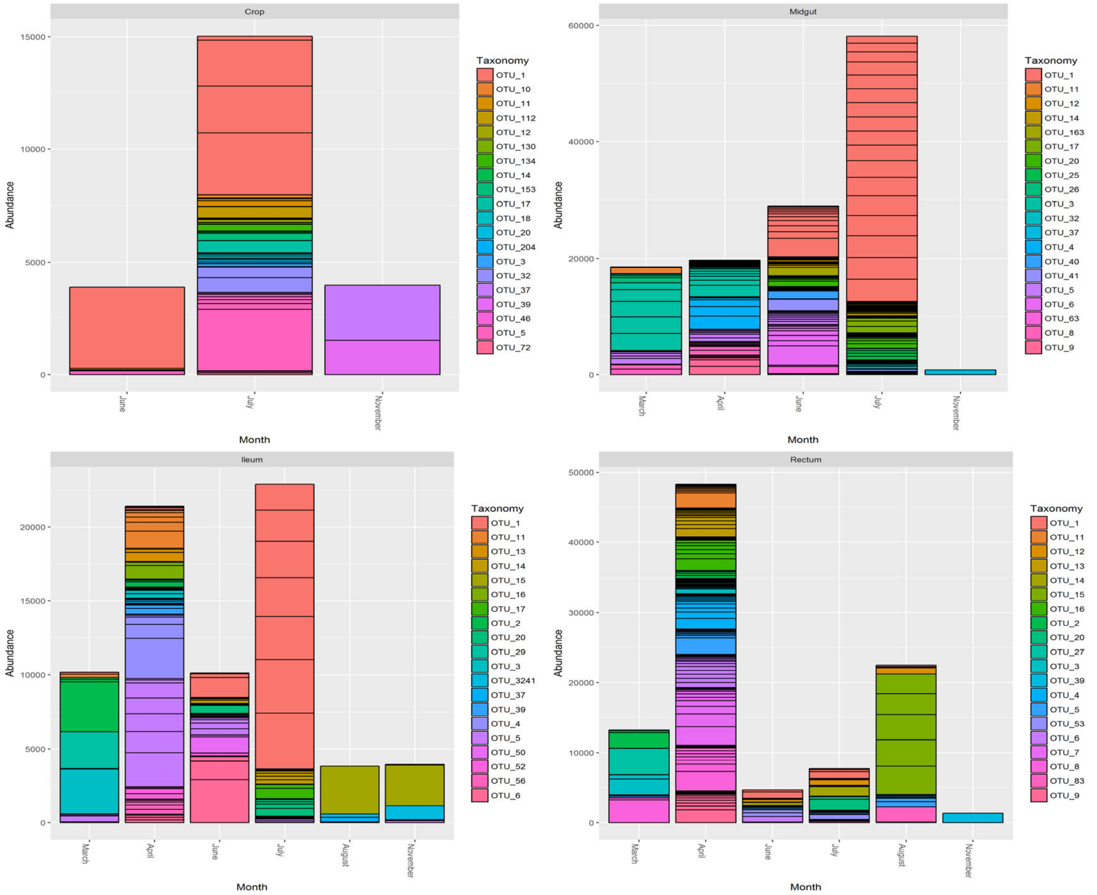

Figure S2. Relative abundance of the 20 most abundant fungal OTUs across months per gut part. Taxonomic assignments are provided in Table S1.

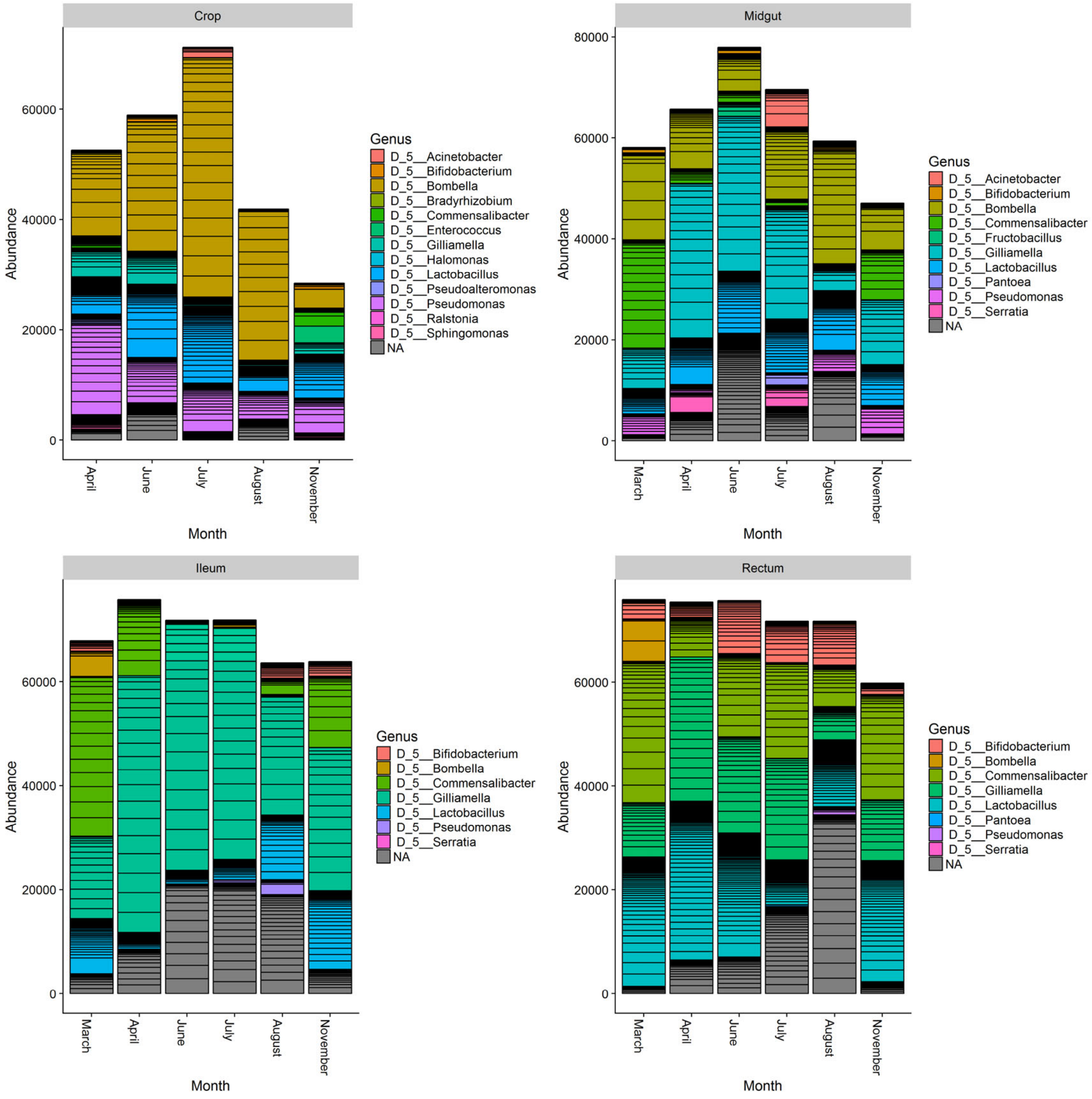

Figure S3. Relative abundance of the bacterial OUTs (with abundance > 1%) across months per gut part. The color code is given at the genus level.

Table S1. Taxonomic assignments of fungal OTUs by Blast.

OTU#	Taxonomy by blast nt collection 11/11-2020	Phylum	Accesion#
OTU_1	Aureobasidium pullulans	Ascomycota	MW085051
OTU_2	Meyerozyma guilliermondii	Ascomycota	MT988167
OTU_3	Starmerella apicola	Ascomycota	KY101940
OTU_4	Debaryomyces hansenii	Ascomycota	MW051606
OTU_5	Engyodontium sp.	Ascomycota	MN905797
OTU_6	Mrakia sp.	Basidiomy-cota	MT505696
OTU_7	Tausonia pullulans	Basidiomy-cota	MN900123
OTU_8	Unknown		
OTU_9	Unknown		
OTU_10	Penicillium corylophilum	Ascomycota	MT906500
OTU_11	Penicillium sp.	Ascomycota	MT993349
OTU_12	Cladosporium sp.	Ascomycota	MW077705
OTU_13	Phaffia rhodozyma/Xanthophyllomyces dendro-rhous	Basidiomy-cota	KY104501/DQ904243
OTU_14	Filobasidium wieringae	Basidiomy-cota	MN899199
OTU_15	Hanseniaspora uvarum	Ascomycota	MN556596
OTU_16	Mrakia gelida	Basidiomy-cota	MN460370
OTU_17	Uncultured fungus clone		MK717974
OTU_18	Cladosporium allicinum	Ascomycota	MT974153
OTU_20	Taphrina carpini	Ascomycota	MK782181
OTU_25	Papiliotrema flavescens	Basidiomy-cota	MN427970
OTU_26	Unknown		
OTU_27	Skoua fertilis	Ascomycota	LR585992
OTU_29	Talaromyces sp.	Ascomycota	LT906538
OTU_32	Curvibasidium cygneicollum	Basidiomy-cota	KY102979
OTU_37	Malassezia restricta	Basidiomy-cota	KX192381
OTU_39	Cladosporium dominicanum/Malassezia globosa	Basidiomy-cota	MW157775/MH093757
OTU_40	Monilinia laxa	Ascomycota	MT156091
OTU_41	Claviceps purpurea		MF462230
OTU_46	Taphrina padi	Ascomycota	AF492104
OTU_50	Neocucurbitaria acerina	Ascomycota	MN251058
OTU_52	Aspergillus conicus	Ascomycota	MN898503
OTU_53	Mucor hiemalis	Zygomycota	MT514370
OTU_56	Penicillium adametzii	Ascomycota	MT601880
OTU_63	Crocicreas amenti	Ascomycota	FJ005092
OTU_72	Uncultured Ascomycota	Ascomycota	FR682219
OTU_112	Entomophthora sp	Zygomycota	GQ285871
OTU_130	Vishniacozyma victoriae	Basidiomy-cota	MN897983
OTU_134	Entomophthora grandis	Zygomycota	GQ285863
OTU_153	Melampsora sp.	Basidiomy-cota	KF780843
OTU_163	Zygosaccharomyces mellis	Ascomycota	AY046190
OTU_204	Curvibasidium rogersii	Basidiomy-cota	JX188232
OTU_3241	Hanseniaspora uvarum	Ascomycota	MH595382
